# Stem cell models of Alzheimer’s disease: progress and challenges

**DOI:** 10.1186/s13195-017-0268-4

**Published:** 2017-06-13

**Authors:** Charles Arber, Christopher Lovejoy, Selina Wray

**Affiliations:** Department of Molecular Neuroscience, UCL Institute of Neurology, 1 Wakefield Street, London, WC1N 1PJ UK

**Keywords:** Alzheimer’s disease, Induced pluripotent stem cells, Neuronal differentiation, 3D cerebral organoids

## Abstract

A major challenge to our understanding of the molecular mechanisms of Alzheimer’s disease (AD) has been the lack of physiologically relevant in vitro models which capture the precise patient genome, in the cell type of interest, with physiological expression levels of the gene(s) of interest. Induced pluripotent stem cell (iPSC) technology, together with advances in 2D and 3D neuronal differentiation, offers a unique opportunity to overcome this challenge and generate a limitless supply of human neurons for in vitro studies. iPSC-neuron models have been widely employed to model AD and we discuss in this review the progress that has been made to date using patient-derived neurons to recapitulate key aspects of AD pathology and how these models have contributed to a deeper understanding of AD molecular mechanisms, as well as addressing the key challenges posed by using this technology and what progress is being made to overcome these. Finally, we highlight future directions for the use of iPSC-neurons in AD research and highlight the potential value of this technology to neurodegenerative research in the coming years.

## Background

It is estimated that 46.8 million people worldwide are living with dementia, a figure that is predicted to increase to 131.5 million by 2050 [1]. Alzheimer’s disease (AD) is the most common cause of dementia, representing an estimated 60–80% of total cases. Pathologically, AD is characterised by the presence of extracellular amyloid plaques composed of the amyloid beta (Aβ) peptide and intracellular neurofibrillary tangles composed of the microtubule associated protein tau. Understanding the contribution of these two pathologies to disease aetiology and neuronal death has been a focus of intense research efforts. However, our progress in this area has been limited by the difficulties in generating in vitro and in vivo models that recapitulate these pathologies together with neuronal death. For example, rodent models generally rely on the overexpression of one or more genes associated with AD and although they can successfully recapitulate some of the disease pathologies, this occurs in the absence of neurodegeneration [2]. Thus, physiologically relevant models, permitting the study of disease mechanisms in a human genetic context, with physiological gene expression levels and in the cell types specifically affected in disease would be a great asset for deepening our understanding of the molecular mechanisms of AD and the development of novel therapeutics.

**Fig. 1:**
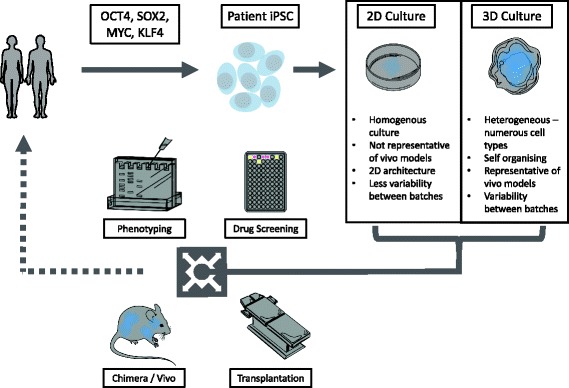
Stem cell applications. Schematic to depict the potential applications of stem cell technology. Patient material is reprogrammed to iPSCs via the four reprogramming genes. Relevant advantages and disadvantages of the subsequent differentaition to cortical neurons via 2D and 3D methods are discussed. Finally, applications for stem cell-derived neurons are depicted, namely drug screening, investigating disease mechanisms, in vivo transplantation of neuronal material to model organisms as well as the potential for cell therapy

Induced pluripotent stem cells (iPSCs) offer an attractive methodology to overcome these barriers in order to generate physiologically relevant models of neurological disease. In 2007, the exogenous introduction of a cocktail of pluripotency-associated transcription factors such as OCT4, KLF4, SOX2 and c-MYC into terminally differentiated cells such as fibroblasts was shown to be sufficient to revert them to a pluripotent state, indistinguishable from human embryonic stem cells (hESCs) [3, 4]. The resulting iPSCs can subsequently be differentiated into multiple cell types, including neurons. The generation and neuronal differentiation of iPSCs from patients with dementia therefore offer a unique opportunity to create physiologically relevant in vitro models for mechanistic studies and preclinical drug discovery, and have been widely adopted by the field (Fig. 1) [5]. In this review, we discuss the progress made to date in using this approach to model AD and highlight the challenges facing the field moving forward.

### Differentiation of iPSCs into relevant cell types

For successful disease modelling using iPSCs, it is essential to generate the cell type affected by disease in a quick and efficient manner with a high degree of purity. The tissues affected early in AD include the neocortex and hippocampus, primarily pyramidal, glutamatergic neurons [6]. Additionally, there is evidence that pyramidal cell innervation by cholinergic neurons is lost early in AD, defining the basis of the cholinergic hypothesis of AD and suggesting that cholinergic cells initiate AD [7]. From the neocortex and hippocampus, tau pathology is seen to spread in a defined manner that traces neuronal connectivity and tracks disease severity [8]. Aβ accumulation focuses in the isocortex and its progression is less predictable than tau (reviewed in [9]). For this reason, the major goal of stem cell differentiation in AD research is the generation of glutamatergic, cortical neurons.

#### Two-dimensional adherent neuronal cultures

Directed differentiation of stem cells towards disease-relevant neurons has gained momentum over the past decade and protocols now routinely generate cultures with high purity of the neuron of interest. Since the first reports of neural specification [10, 11], information about extrinsic signalling conditions gleaned from developmental biology have been applied to stem cell differentiation, leading to defined fate choices and increasingly pure neuronal populations (reviewed by [12]). With this in mind, stem cell differentiation closely mirrors developmental events, timing and signalling conditions—akin to applied developmental biology.

Stem cell neurogenesis progresses through three phases: neural induction, patterning, and terminal differentiation. In 2D cultures, dual SMAD inhibition has been widely adopted to enhance neural fate choice and closely mimic signalling conditions in the early embryo [13, 14]. Following neural commitment, human neuroepithelium has a default differentiation potential to become cortical tissue. This is due to an anterior neural character with high levels of Wnt signalling, leading to dorsalisation of the cells [15]. This provides a great efficiency to cortical differentiation protocols and can be further enhanced by inhibiting ventral fates via Sonic hedgehog (SHH) antagonists [16]. At this stage, extrinsic signalling conditions can be manipulated to lead to differentiation to other cell types of interest [15]. Committed neural progenitors are subsequently allowed to undergo protracted terminal differentiation to mature neurons, a process that follows in vivo corticogenesis. Protocols that are widely employed in the field follow these defined milestones [14, 17, 18] and have been shown to be reliant on retinoid signalling [18] and also are able to produce cells that functionally integrate with host circuitry after transplantation into rodent models [17].

The cortex is made up of six defined neuronal layers with characteristic identity and marker expression. In vivo, these are generated in a time-dependent manner, with deeper layers generated first and upper layers later. Inhibitory interneurons migrate from ventral tissues into the cortex, modulating the activity and development of cortical excitatory neurons. These processes are all recapitulated by differentiating stem cells in vitro, whereby upper layer neurons are generated later in culture, necessitating differentiation periods of 100 days in vitro to generate a full complement of electrically active neurons [17, 18].

Recent reports have attempted to accelerate stem cell differentiation, making the in vitro model more accessible. This has been achieved by either overexpression of the pro-neural transcription factor NGN2, which generates homogeneous cortical populations in 2–3 weeks [19] or a combination of small molecule inhibitors targeting ERK, FGF, Notch and Wnt signalling pathways, which leads to a highly pure population of functionally active deep-layer neurons in just 13 days [20].

In addition to cortical glutamatergic neurons, protocols for the directed differentiation of stem cells into other cell types relevant to AD have been developed. These include cortical interneurons [21, 22] and cholinergic neurons [23], two cell types that have been suggested to be involved in AD. Both cell types develop from the ventral forebrain and so are dependent on ventralising signals such as SHH. As in vitro models increase in complexity, such as investigating the interplay between neuronal subtypes, the employment of multiple models will become increasingly relevant.

After extended periods in culture, neurogenic cultures undergo a gliogenic switch, producing primarily astrocytes rather than neurons [24]. Treatment with mitogens, such as FGF, and patterning molecules, such as BMP and LIF, leads to increasingly enriched astrocyte cultures [25]. Astrocytes provide important support to neurons and are increasingly implicated in non-cell autonomous disease processes. These will be discussed in detail below.

The differentiation of stem cells in two dimensions benefits from consistent extracellular conditions throughout the culture, whereby all cells are exposed to similar growth factor concentrations throughout the adherent monolayer, giving great control to the user and high levels of purity of the resultant population. This is relevant not only for extrinsic growth factors but also for applications such as genetic manipulation or drug screening. The resultant cultures display electrophysiologically active neurons, vital for studying AD processes in the cell-relevant model. However, these cultures represent a reductionist approach and the small number of cell types may be seen as a drawback. Additionally, iPSC-derived neurons have a foetal developmental age [26, 27], necessitating careful considerations when investigating ageing diseases such as AD.

#### Three-dimensional *neuronal cultures*

In an attempt to better model the developing brain, rigid scaffolds have been employed to produce 3D neuronal cultures. These are created from a variety of materials, including, but not limited to, polydimethylsiloxane with micrometric cavities [28], super-porous/non-porous hydrogels, sintered titanium [29] and hydrogels or matrigels [30]. Mixtures of microfibre and nanofibre scaffolds made from polymers such as poly lactic acid and polyethylene terephthalate, respectively, enable structural integrity yet small pore size to increase cellular adhesion [31].

Primary neurons and differentiated iPSCs have been used in a 3D soft matrix scaffold, conferring some advantages over 2D. Neuron bodies are more rounded and in proximity with cells on all sides and neurites can extend in all directions, closely modelling the axonal architecture in vivo [28, 32].

The aforementioned support structures accomplish 3D tissue culture in different ways. Ridged and inorganic scaffolds serve more as support and guidance for cellular growth, while softer encapsulating scaffolds mimic the extracellular matrix, providing support as well as promoting growth within itself [28, 33]. However, these 3D cultures are more akin to a cell suspension within a supportive matrix, rather than a fully 3D tissue.

#### Three-dimensional *organoid cultures*

Although 2D cultures and scaffolds offer the benefits outlined above, they lack the cellular diversity, structural complexity and physical architecture seen in vivo. Thus, efforts to create 3D models of neurons and glia are crucial for developing physiologically relevant models [34].

Neural differentiation in three dimensions follows the same three processes as adherent monolayer protocols, namely neural induction, patterning and terminal differentiation. Upon exit from the pluripotent state, the small size of the 3D structures, termed embryoid bodies (EBs), enable the researcher to have close control over the neural fate choice. Similar to 2D cultures, SMAD inhibitors may be used at this stage to enhance neural fate choice [14, 35, 36].

In contrast to differentiation in 2D, however, 3D strategies allow intrinsic cell behaviour to determine the development of the EB or organoid. Cell autonomous signals lead to events such as migration, polarisation of neuroepithelium and generation of a range of cell subtypes, culminating in self-organised heterogeneous neuronal tissue. Spatiotemporal signalling events and inherent gene cascades, with inherent genetic competencies and responses tightly controlled in time and space, strictly govern these complex cell behaviours. This self-organising phenotype gives 3D suspension organoids more in vivo relevance than the scaffolded 3D tissue cultures discussed above.

Organoids have been shown to exhibit defined radial glial cells, a cell type relevant to brain development and higher function. These show appropriate morphology and organisation, being representative of the developing human cortex [37]. Supportive cells that develop alongside early neurons may well be vital in modelling disease initiation and progression [38]. These events enable complex experimental questions to be asked, such as the cross-talk between cell types, e.g. neurons and glia. This is a further advantage for drug discovery when support cells may be crucial or the pathway of interest is not known [34]. The fact that a 3D organoid more closely resembles tissue goes some way to reducing 2D cell culture artefacts and improving the robustness of findings in 2D cultures.

The development of organoids patterned towards the cortex has progressed in recent years. Endogenous patterning and terminal differentiation produce 3D structures with appropriate cortical cell layers and functional neurons [39]. These structures can also be further patterned to other brain regions of interest by the addition of extrinsic growth factors, albeit the effects are seen at the outer portions of the 3D structure [37, 40, 41].

In spite of these advances, organoids also have several limitations. The self-organising nature by which organoids are generated leads to increased heterogeneity between batches [37]. This variability between organoids means analysis methods such as bulk-population transcriptomics or western blotting on whole tissue extracts may be difficult to interpret. However, single-cell transcriptomics has successfully shown that the diversity of cell types present in organoids closely resembles that present in developing foetal brain [27]. This provides confidence in the physiological relevance of the system but once again raises the issue of developmental maturity and how relevant cerebral organoids are in representing the modelling of late-onset diseases. Finally, when organoids exceed 6–10 mm in diameter, the inability of nutrients to penetrate the organoids results in a necrotic centre, leading to the release of caspases and other detrimental cell signals, potentially increasing cell stress in larger organoids [42]. Organoids have been successfully kept in continuous culture for up to one year, although some shrinkage can be observed from 6 months onward, when the necrotic core usually starts to develop [43]. To halt proliferation of the organoid, reducing the chance of a necrotic core developing, inhibition of mitogenic signals such as Notch can increase terminal differentiation, which may enhance maturity [44, 45].

On a practical level, culture of cerebral organoids within spinning flasks [46] has been shown to offer enhanced diffusion of nutrients and oxygen, allowing for larger and more continuous organoid growth. However, these large custom-made organoid systems are expensive to purchase and require large amounts of costly media. The development of 3D-printed bioreactors provides a compact and economical method for organoid culture; 3D-printed bioreactors are plastic lids for standard six-well plates that incorporate mini-stirrers attached to an electric motor, allowing constant media movement around the organoids. This enables users to easily adopt them into existing cell culture facilities without the requirement of accommodating large orbital shakers or spinning bioreactors in tissue culture incubators [37]. Further, mini-bioreactors reduce heterogeneity in organoids and reduces the amount of media required, reducing the costs associated with organoid generation [47].

In spite of these drawbacks, organoids have been used very successfully to model neurodevelopmental processes and diseases, for example normal cortical folding [48], microcephaly [30] and lissencephaly [49]. Importantly, organoids have been used to uncover species-specific differences between human and murine cortical development that lead to folding and expansion in human tissues specifically [48]. Although reports of organoid models of neurodegenerative disease are limited at present, they have the potential to be an insightful new model in AD research, allowing researchers to experiment with more heterogeneous, naturally organised 3D cell models.

### iPSC-neurons in AD research

Much of what we know about the molecular mechanisms of AD has been guided by our knowledge of the genetics of disease and specifically autosomal dominant, familial AD (fAD). Pathologically, AD is characterised by the presence of Aβ plaques and neurofibrillary tangles. Studies into autosomal dominant AD identified mutations in amyloid precursor protein (APP), Presenilin 1 (PSEN1) and Presenilin 2 (PSEN2) that are causative of disease, although 99% of AD is sporadic. The commonalities in pathology between fAD and sporadic AD (sAD) imply that studies in fAD can be broadly applicable to AD as a whole.

APP, PSEN1 and PSEN2 have been shown to function in a single pathway, namely APP processing, centrally implicating this pathway in AD pathogenesis. APP is a transmembrane protein with unknown function present in endosomes and at the cell surface. Sequential cleavage by either α-secretase and γ-secretase or β-secretase and γ-secretase leads to extracellular soluble APP (sAPP), which may function in cellular signalling, an intracellular domain with possible cell-autonomous functions, and small membrane fragments. The β-secretase and γ-secretase path produces Aβ, a small peptide that was shown to be the primary constituent of amyloid plaques.

PSEN1 and PSEN2 are alternative subunits of the γ-secretase complex. Thus, alterations in APP processing and Aβ imbalance are thought to lie behind AD. Much is still to be understood about the normal function of the γ-secretase complex (known to cleave multiple substrates such as Notch, Cadherin and Ephrin as well as APP) as well as the different specificities and expression patterns of its components. For example PSEN1 localises throughout the cell whereas PSEN2 is restricted to the endosome and lysosome compartments [50].

The following section discusses the relative promise and early successes in the use of iPSC-derived cells to model AD pathology and offer insight into disease mechanisms.

### Investigating the normal function of AD-associated proteins in human iPSC-neurons

Human iPSC-derived neurons represent a useful tool for studying the normal function of AD-associated proteins in a human neuronal setting. For example, the function of APP is yet to be fully understood, with complex splice variants, cell compartmentalisation and cell specificity (reviewed by [51]). sAPP, the soluble fragment of APP shed from the neuron after cleavage by α or β secretase, induces a strong pro-differentiation effect towards the neural lineage from hESCs [52, 53]. Premature differentiation is also seen in iPSC-derived neural progenitors transfected with mutant transgenic PSEN1 [54]. The Notch and Wnt signalling pathways, which regulate neuronal development among other cellular processes, are involved in aberrant differentiation of these mutant PSEN1 lines.

In an elegant study, Liao and colleagues used single cell techniques to understand the sAPPα and Aβ profiles of individual iPSC-neurons and iPSC-astrocytes [55]. fAD mutations did not alter the overall profiles of sAPPα and Aβ across the population. Critically, there was no bias in Aβ secretion towards neurons or astrocytes; however, trends to increased secretion were present in deep layer cortical cells and GABAergic interneurons.

These studies have applied iPSC technology to gain new knowledge of APP and PSEN1 function in human neurons. The physiological relevance of the cell model enables new insights into the normal functions of these proteins in neurons. As well as these studies into the normal role of APP and PSEN1 in human iPSC-neurons, a wide variety of investigations are making use of iPSC technology. To exemplify the breadth of the model’s application, two examples include screening for modulators of APP processing in iPSC-derived neurons [56] and iPSC-derived macrophage like cells overexpressing the Aβ degrading enzyme Neprilysin 2 that are able to reduce Aβ levels in culture media and the rodent brain [57]. The scope of research into AD using iPSC-derived neurons will be discussed in the sections below.

### Investigating aggregate toxicity using iPSC-neurons

Stem cell-derived neurons have been used to investigate cellular toxicity of Aβ oligomers [58, 59]. Employing cortical neurons generated from control iPSCs enables investigations into downstream signalling and toxicity induced by exogenous AD-associated peptides in human neurons for the first time.

Aβ was shown to bind to glutamatergic and GABAergic iPSC-derived neurons but specifically led to cell death in glutamatergic neurons [58]. Eight days of oligomeric Aβ treatment was shown to be sufficient to lead to synaptotoxicity [59]. This was demonstrated via altered axonal vesicle clusters, impaired postsynaptic AMPA signalling and a resultant induction of phosphorylated tau and endoplasmic reticulum (ER) stress. It should be noted that the concentrations of Aβ sufficient to induce cell death in these models (1–5 μM) are in excess of normal physiological levels, which are believed to be in the low nanomolar range [60] in control tissue and increased up to 50 nM in AD brain tissue [61].

Together, these studies have reinforced investigations using cell lines and rodent models which demonstrate cellular toxicity resulting from pathological spread in AD. Synaptic loss and calcium imbalance in healthy human neurons in response to AD-associated aggregates may hint at early pathological mechanisms.

### iPSC models of fAD

The reprogramming of patient-derived cells to neurons allows cultures of human neurons with genetic predisposition to AD to be probed for the earliest disease events. Not only can the direct effects of enzyme mutations in fAD be used as a readout, but also sporadic disease with undefined genetic causes or risks can be modelled. iPSC-derived neurons are being widely employed to study the pathological processes in AD, the spread of pathology and also the earliest events underlying disease aetiology (Fig. 1).

#### APP processing

Several studies have now demonstrated that fAD patient-derived iPSC-neurons display an increased Aβ42:40 ratio and an increase in phosphorylated tau compared to control iPSC-neurons (Table 1) [62–76]. These features have also been explored in sporadic patient-derived cells with varied results as some lines behave as fAD and some as control lines with respect to Aβ secretion and tau phosphorylation [64, 67]. The majority of these studies have been based on cortical differentiation protocols, necessitating long differentiation periods as APP expression and Aβ peptides progressively increase from 30 days of differentiation to mature cultures at 100 days [63, 71, 77].

**Table 1:** Summary of studies using genomically unaltered iPSC-derived neurons to investigate AD

Study	Mutations investigated	Aβ	Tau	γ-Secretase inhibitor	β-Secretase inhibitor	Other drugs	
Yagi et al. 2011 [62]	PSEN1 A246E PSEN2 N141I	↑Aβ42 ↑Aβ42:40		Compound E ↓Aβ		Compound W ↓Aβ42	• New insights into AD
Yahata et al. 2011 [63]	Controls			GSI ↓Aβ	BSI ↓Aβ	NSAID ↓Aβ	• Relevant expression of APP and secretase subunits in iPSC-neurons
Israel et al. 2012 [64]	APP duplication sAD	↑Aβ40	↑pTau ↑Activated GSK3β				• Swollen endosomes • sAD variability
Shi et al. 2012 [65]	DS	↑Aβ40, ↑Aβ42 Aggregates	↑pTau and redistribution	DAPT ↓Aβ			
Kondo et al. 2013 [67]	APP E693Δ→ APP V717L→ sAD	↓Aβ40 ↑Aβ42 Aβ oligomer accumulation			↓Aβ	DHA ↓Aβ	• Difference effect of two mutations • Intracellular Aβ • UPR and ROS
Woodruff et al. 2013 [76]	PSEN1 ΔE9	↑Aβ42:40		Compound E ↑Aβ42:40 of wild-type neurons			• PSEN1 ΔE9 is dominant negative and does not affect γ-secretase-independent functions
Duan et al. 2014 [68]	PSEN1 A246E fAD APOE	↑Aβ42:40		Compound E ↓Aβ40			• APOE4 cells are sensitive to neurotoxic stimuli
Muratore et al. 2014 [71]	APP V717I	↑Aβ42:40		DAPT ↓Aβ			• APP mutant cells ↑APP (protein and not RNA), ↑tTau, ↑pTau
Mahairaki et al. 2014 [69]	PSEN1 A246E	↑Aβ42:40 ↑sAPPβ		DAPT ↓sAPPβ			• Presence of APP in endosomes
Sproul et al. 2014 [72]	PSEN1 A246E PSEN1 M146L	↑Aβ42:40					• Altered gene pathways include NLRP2, ASB9 and NDP
Chang et al. 2015 [74]	DS	↑A40 ↑A42 Aggregates	↑tTau ↑pTau	DAPT ↓Aβ, ↓tTau ↓pTau		Bdph ↓Aβ, ↓tTau, ↓pTau	• Tau mislocalisation
Moore 2015 [75]	APP V717I APP duplication PSEN1 ΔI4 PSEN1 Y115C DS	↑A42:40 APP dup unchanged	APP (not PSEN1) leads to ↑tTau ↑pTau	DAPT and E2012 γ-Secretase and APP are coupled to Tau	LY2886721 does not alter Tau		• APP processing and intracellular fragment linked to Tau proteostasis
Hossini 2015 [153]	sAD		↑pTau, ↑activated GSK3β	Compound E			• Neurodegenerative gene pathways downregulated
Raja 2016 [78]	APP duplication PSEN1 M136I PSEN1 A 264E	↑Aβ Aggregates	↑pTau	Compound E ↓Aβ and ↓pTau	BACE-1i ↓Aβ and ↓pTau		

*Abbreviations*: *Bdph* N-butylidenephthalide, *BSI* β-secretase inhibitor, *DHA* docosahexaenoic acid, *DS* Down’s syndrome, *GSI* γ-secretase inhibitor, *NSAID* non-steroidal anti inflammatory drug, *pTau* phosphorylated Tau, *ROS* reactive oxygen species, *sAPP*β soluble APP (β-secretase pathway), *tTau* total Tau, *UPR* unfolded protein response

In addition to monogenic fAD models, APP dosage models have been shown to demonstrate similar changes in APP processing to patients with APP duplication [64, 78] and Down’s syndrome (trisomy of chromosome 21 on which APP resides) [65, 74].

#### Drug discovery

iPSC technology offers a unique opportunity for drug discovery. Not only can efficacy and toxicity studies be carried out in cells such as neurons and hepatocytes, but also reporter cell lines can be used to screen for drugs with desired responses from large compound libraries. For example, cells that express GFP under the NKX2.1 promoter have been employed to develop efficient differentiation protocols to generate cortical interneurons, a cell type dependent on NKX2.1 expression. Finding small molecules that enhance GFP cell numbers can then replace costly recombinant protein growth factors, as such the WNT antagonist XAV939 [22]. With respect to AD, the accepted link between increased Aβ42:40 ratio and dementia may become the basis of future drug discovery platform readouts. A proof of concept for this has been shown as γ-secretase inhibitors are able to reduce total Aβ levels and Aβ42:40 ratios (Table 1) [58, 64, 69, 71, 79]. These models can potentially reduce the load on animal efficacy studies while also representing a human-specific model.

Similarly, β-secretase inhibitors have been shown to reduce total levels of Aβ [63, 67] and may influence GSK3β activity and tau phosphorylation more directly than γ-secretase inhibitors [64]. Direct inhibition of GSK3β (and so activation of Wnt) leads to reduced levels of phosphorylated tau without affecting Aβ accumulation [79]. A phthalide compound that also increases Wnt signalling had a similar effect on tau and also reduced Aβ levels in Down’s syndrome iPSC-neurons [74]. Longer treatments of β-secretase inhibition in 3D models was shown to reduce both phosphorylated tau and Aβ [78].

The effects of other drug molecules have been investigated using fAD iPSC-derived neurons. Compound W, a specific Aβ42 inhibitor, was shown to reduce the Aβ42:40 ratio [62]. Non-steroidal anti-inflammatory drugs (NSAIDs) are also able to reduce the Aβ42:40 ratio [63], although some PSEN1 mutations seem unresponsive to NSAIDs [66].

These studies demonstrate the potential utility of iPSC-neurons to provide a neuronal platform for toxicity or efficacy screening that may highlight potential candidate molecules, although critical hurdles remain. Firstly, off-target side effects are not evident in neuronal cell cultures, and secondly, a drug’s ability to cross the blood–brain barrier is not addressed in reductionist models consisting of pure cell populations. However, the use of this platform to narrow a drug pipeline can undoubtedly save pharmaceutical companies time and money as well as increase the likelihood of finding a potential hit. As an example of the potential success and value of the technology, iPSC models of familial disautonomia have led to clinical trials following small molecule screens in vitro [80].

#### Biochemical effects of fAD mutations

The biochemical mechanisms behind the change in Aβ ratio have been investigated using iPSC-neurons. The A673T mutation in APP leads to a reduced likelihood of contracting AD [81]. Using iPSCs and their isogenic controls, Maloney and colleagues demonstrated that this mutation leads to a reduced efficiency of β-secretase cleavage of APP and a subsequent reduction in the ability of mutant Aβ to form aggregates [70]. This result reinforces the central role of Aβ in disease.

In contrast, utilising the V717I mutation in APP, Muratore et al. [71] were able to show that mutant APP had an increased likelihood to be cleaved by β-secretase over α-secretase and therefore Aβ accumulated. Blocking antibodies to Aβ were able to prevent the Aβ-associated tau pathology in these cells, directly linking extracellular Aβ to downstream tau phosphorylation.

The link between APP processing and tau has been further investigated in iPSC-derived neurons. APP mutations, but not PSEN1 mutations, were shown to directly lead to increased total tau and phosphorylated tau in iPSC-derived neurons [75]. Extended periods of γ-secretase modulation were able to reverse these features and hint that APP processing can influence tau in an Aβ-independent manner.

Woodruff and colleagues made use of gene-editing technology in iPSCs to investigate the precise effect of dominantly inherited PSEN1 mutations in human neuronal cultures without overexpression [76]. The authors demonstrated that Δ exon 9 PSEN1 (ΔE9) mutations lead to reduced γ-secretase activity, shown via an accumulation of unprocessed APP C-terminal fragments and altered Aβ42:40. Importantly, these effects were seen in wild-type/ΔE9 cells and ΔE9/null cells, but not wild-type/null cells. These results point towards a toxic gain of function via poisoning of the γ-secretase complex by mutant PSEN1 protein. Interestingly γ-secretase-independent functions of PSEN1 remain unchanged [76], shown via the ability of cells to produce mature Nicastrin [82]. It should be noted that some controversy remains about whether PSEN1 mutations are gain or loss of function (see the discussion in Woodruff et al. [76]).

#### Novel insights into molecular mechanisms

Israel et al. [64] described aberrant swollen early endosomes in the soma of AD-derived neurons. Further, Aβ was shown to accumulate in the ER, endosomal and lysosomal compartments, leading to ER stress and reactive oxygen species (ROS) damage [67, 71]. In E693Δ APP mutant neurons, which show intracellular Aβ accumulation, ER and oxidative stress were shared between AD neurons and astrocytes and reversed by drugs including γ-secretase inhibitors and docosahexaenoic acid (which did not alter Aβ levels) [67]. These studies implicate intracellular Aβ as an important disease modulator and potentially the endosome as a central compartment for APP proteolysis, a compartment to which PSEN2 and only fAD-mutant PSEN1 localise [50].

Similar cellular pathways were shown to be affected in sAD patient-derived neurons; a reduction was observed in the unfolded protein response, oxidative damage and cell death pathways (pathways common to other neurodegenerative diseases) whereas Wnt signalling pathways were induced [67]. Interestingly, pre-existing Aβ aggregates were degraded after inhibition of β-secretase, suggesting cells retain an undefined ability to degrade accumulated Aβ. Removal of pre-existing aggregates may be crucial to the ability of inhibitors to reverse cellular stress responses and provide insights into disease and treatment mechanisms.

The downstream mechanisms resulting from fAD mutations have also been analysed. iPSC-derived neural progenitors were shown to have a number of differentially expressed genes when compared with healthy control cells [72]. Encouragingly, five of these genes were also dysregulated in post-mortem brain of AD patients. These similarities show the relevance of the in vitro models, but also can highlight molecular pathways that may lie upstream of pathological processes in non-aged neurons. These include Norrin, a molecule involved in Wnt signalling and shown by the authors to lead to reduced neurogenesis in the AD brain [72].

In sum, these studies have successfully employed iPSC-derived neurons to provide new insights into AD (summarised in Table 1).

### Investigating AD risk factors using iPSC-neurons

In addition to autosomal dominant mutations in PSEN1, PSEN2 and APP, multiple genetic variants that lead to increased risk of late onset AD have been identified. The APOE4 variant was initially identified in 1993 as an AD risk factor [83]. Genome-wide association studies in large cohorts have enabled the identification of multiple loci associated with increased likelihood of AD. These include CLU, CR1 and PICALM [84] and SORL1 [85] amongst others [86–88]. Heterozygous mutations in TREM2 were shown to increase risk of AD by one-third [89, 90]. Importantly, the cellular mechanisms linking these genetic loci to increased AD risk remain to be understood. Pathways such as inflammation and the endosome/lysosome pathway have single nucleotide polymorphisms (SNPs) in multiple genes implicated in AD risk, suggesting a central function of these pathways in disease progression.

Patient-derived iPSC-neurons enable investigations into the effects of risk-associated SNPs and mutations. To date, four articles have described investigations into AD risk factors utilising iPSCs derived from SNP carriers in APOE, SORL1 and CR1 [68, 91–93].

Basal forebrain cholinergic neurons (BFCNs) have been shown to degenerate early in AD pathogenesis [94]. Duan et al. [68] generated BFCNs from fAD and sporadic patients with APOE3/E4 risk genotypes. All AD lines showed increased Aβ42:40 and glutamate-induced vulnerability. Interestingly, one sporadic line showed increased Aβ40 secretion at low concentrations of γ-secretase inhibition. This is reminiscent of the variability witnessed in other studies at low doses of inhibition [76] or alternatively may be mutation-dependent [66].

The link between APOE genotype and APP was further investigated using ESC-derived neurons by Huang et al. [92]. The authors show that APOE stimulates APP production in neurons, and subsequently Aβ, via MAPK signalling. The AD risk factor APOE4 stimulates more APP and Aβ production than the common allele APOE3, and the protective allele APOE2 induces the lowest APP protein production [92]. This provides an intriguing link between the role of APOE, a lipid transporter, and the initiation of pathology. Mouse models also hinted that APOE has an effect through disrupting mitochondrial respiration, specifically in neurons [95].

SORL1 is also an AD risk factor, with homozygosity of risk variants showing increased risk for disease progression. Young et al. [91] investigated the effects of SORL1 variants on neurotrophin response in iPSC-derived neurons. Possession of a risk genotype abrogated the induction of SORL1 expression in response to brain-derived neurotrophic factor (BDNF). This lack of induction of SORL1 expression further led to no change in Aβ40 in response to BDNF, whereas Aβ40 was reduced in control cells. This study therefore places SORL1 as a crucial intermediate between BDNF signalling and reduced Aβ expression, a pathway inhibited by SORL1 risk SNPs.

Stem cells have been generated from patients with copy number variations (CNVs) for the risk factor CR1 (complement receptor 1) and, based on CR1 function in the immune system, it will be interesting to learn the consequences of CR1 CNVs in differentiated cell types, such as immune cells versus neurons [93].

These studies demonstrate the power of stem cell techniques, whereby known genetic risk factors can be investigated, but also undefined mutations and risk factors can be modelled from patients with sporadic disease.

### Three-dimensional iPSC-neuronal models of AD

The use of 3D iPSC-derived organoids is now being realised for research into dementia.

From a pathological perspective, 3D neuronal tissues have internal ventricles as well as many interneuronal spaces, something 2D cultures lack. Further, media changes in 2D cultures will remove proteins secreted by cells, such as Aβ peptides, which will affect their ability to form aggregates. However, organoids or matrix-embedded 3D cultures retain them within their 3D structure, increasing the local concentration of proteins and potentially increasing the likelihood of pathological aggregates [78].

Elevated levels of extracellular Aβ and intracellular phosphorylated tau were demonstrated in neurons differentiated from neural stem cells overexpressing both fAD-associated mutant PSEN1 (ΔE9) and APP (K670N/M671L plus V717I) [79]. Differentiated cells were cultured in either 2D adherent cultures or embedded into matrigel to generate 3D cultures. The authors were able to show Aβ40 aggregates in 3D as well as phosphorylated, insoluble, silver-positive tau aggregates for the first time that were confirmed as tau filaments via electron microscopy. These aggregates were present specifically in 3D cultures and not 2D cultures of the same cells. Treatment of the organoids with β- and γ-secretase inhibitors reduced Aβ and also insoluble tau, and GSK3β inhibitors were able to reduce phosphorylated tau, demonstrating the suitability of 3D models for drug discovery studies.

Raja and colleagues used generated 3D organoids, using a protocol described by Kadoshima and colleagues, to investigate pathological processes in the absence of overexpression in cells from patients with APP duplications and M146I and A264E PSEN1 mutations [96, 78]. Amyloid deposition was apparent after 60 days of culture, with increased levels at day 90. An increase in hyper-phosphorylated tau (pS396) between fAD organoids and controls became apparent after 90 days in culture. Both amyloid deposition and hyper-phosphorylated tau were reduced via treatment with β- and γ-secretase inhibitors [78]. Encouragingly, the enlarged endosome phenotypes witnessed in 2D studies [64, 67, 71] was recapitulated in this study, reinforcing the findings in both paradigms. The development of bona fide neurofibrillary tangles in the absence of overexpression of one or more mutated genes has yet to be described. This is likely due in part to the fact that tangle formation occurs over extended time-periods not reflected by cell culture models. It is also worth considering that cell culture models may not reflect the diversity of tau isoforms seen in the adult brain (discussed further below) and the necessity of this to the development of tau pathology is not known. Further, although tau hyperphosphorylation is a pathological feature, tau is also highly phosphorylated during early development and these fetal-like cells may be resistant to tangle formation [97, 98]. The development of in vitro human models that more accurately recapitulate plaque and tangle pathologies seen at post-mortem will be a major step forward for the field; however, these early-stage models also provide an attractive opportunity to understand the earliest pathological changes to tau and the sequence of events leading to the tangle pathology observed at post-mortem. Further, several groups have now reported a dissociation between tangle formation and neurodegeneration [99, 100], so the absence of tau tangles does not prohibit the study of tau-associated disease mechanisms.

In addition to fAD, sAD patient-derived iPSCs have been used for 2D and 3D models. Treatment with γ- and β-secretase inhibitors was shown to reduce Aβ secretion levels. Interestingly, in one sAD patient no decrease in Aβ42 was seen in response to β-secretase inhibition, hinting at patient variability in sAD. Also, identical inhibitor concentrations showed differing efficacy in 2D and 3D sAD cultures, which has important implications for drug discovery between the two models [101].

In a direct comparison, the phosphorylation and activation of p21-activated kinases (PAKs) in response to exogenous Aβ was witnessed in 3D organoids and not in 2D iPSC-derived neurons. This was in addition to downstream cytoskeletal changes. This finding reinforces the notion that Aβ displays local accumulation in 3D cultures leading to more representative pathological changes [102].

These studies point to the relevance of 3D models of AD, both recapitulating some of the findings from 2D studies as well as showing aggregated tau for the first time (albeit after its overexpression). Organoids can also be employed to investigate complex events that are inaccessible in 2D, for example the spatial relationship between tau and amyloid and the spread of protein conformation through a structure.

### iPSC models of frontotemporal dementia linked to mutations in *MAPT*

Mutations in the tau gene, *MAPT*, are causative for frontotemporal dementia (FTD) with tau pathology. Although mutations in *MAPT* do not cause AD, there is a high level of correlation between tangle burden and disease severity, together with compelling evidence from experimental models demonstrating that tau mediates amyloid toxicity [75]. Thus, a discussion of recent efforts to model tau mutations using iPSC-neurons is warranted here. To date, more than 40 mutations in *MAPT* linked to FTD have been described, the majority of which are clustered around the C-terminal half of the protein, within or close to the microtubule-binding region. Thus, it is hypothesised that mutations may either disrupt the binding of tau to microtubules or promote its propensity to form aggregates, two possibilities that are not mutually exclusive.

#### Tau splicing in iPSC-neurons

The alternative splicing of the *MAPT* gene leads to the production of six protein isoforms of tau, differing by the presence of zero, one or two N-terminal repeats (0 N, 1 N or 2 N) and three or four microtubule binding repeats at the C-terminus (3R or 4R) [103–105]. Tau splicing is developmentally regulated; only the smallest tau isoform (0N3R) is expressed during foetal development, but all six isoforms are expressed postnatally, with approximately equal levels of 3R and 4R tau [103]. Tau splicing is tightly controlled and appears to be critical for neuronal health. A subset of *MAPT* mutations disrupt tau splicing, generally leading to an increase in 4R tau [106, 107]. An over-representation of 4R tau is also observed in the sporadic tauopathies progressive supranuclear palsy and corticobasal degeneration [108]. The mechanisms by which altered tau splicing can lead to disease remain poorly characterised but may relate to an increased propensity of 4R tau to aggregate, or altered microtubule dynamics leading to changes in axonal transport [109].

One important opportunity offered by iPSC-neurons in this regard is their ability to accurately reflect the complex expression and splicing of tau seen in the adult human central nervous system, something that is not recapitulated in rodent and other animal models. However, this has proved to be challenging due to the relative immaturity of neurons derived from iPSCs. Genome-wide transcriptomics studies have demonstrated that iPSC-neurons closely resemble foetal neurons in the context of gene expression profiles [26]. This also holds true for tau expression and splicing, where several studies have demonstrated that 0N3R (foetal) tau is the predominant tau isoform expressed in iPSC-neurons [110–113]. Although after extended in vitro culture periods iPSC-neurons initiate expression of multiple tau isoforms, this appears to require between 150 and 365 days in culture, which is prohibitive for routine experimental use [110, 112]. These findings have important implications for disease modelling. Several coding mutations commonly used for in vitro and in vivo disease modelling, such as P301L and P301S, are located within the alternatively spliced exon 10. Thus, extended culture periods would be required in order for the mutant protein to be expressed at high levels. As discussed below, methods now exist to accelerate in vitro cortical differentiation from 100 days to 16 days [20], and promote accelerated aging in cultured neurons [114, 115], although it has not yet been assessed if any of these approaches will accelerate mature tau splicing. However, transplanting human neurons into mouse brain leads to a rapid maturation and expression of 3R/4R tau at a 1:1 ratio—as observed in adult human brain—at 8 months post-transplantation [116]. Interestingly, these chimaeric models also demonstrate the specific vulnerability of human neurons to Aβ, although it is yet to be determined if this is mediated by tau [116].

In spite of these challenges, numerous reports now describe successful modelling of tauopathy in iPSC-neurons with *MAPT* mutations. Intronic mutations in *MAPT* such as IVS 10 + 14 and 10 + 16, together with coding mutations known to alter tau splicing, such as N279K, are able to override the developmental regulation of tau splicing, leading to the expression of 4R tau isoforms at early time points in vitro in iPSC-derived cortical neurons [110–113]. This appears to have an effect on neuronal function, leading to a more rapid acquisition of electrical maturity [112]. Given that differentiation protocols closely mimic in vivo development, this raises the intriguing question of the relevance of these findings to development in utero. Could neuronal development and functional connectivity be altered even in early development? The early presence of 4R tau also presents with increased cellular stress in N279K neural stem cells, as determined by the increased presence of the stress granule markers G3BP and TIA-1, together with altered intracellular vesicle composition as identified by increased levels of the endosomal markers flotilin-1 and EEA1 [111].

#### Modelling tau coding mutations in iPSC-neurons

Despite the aforementioned challenge of obtaining neurons expressing the mature complement of tau isoforms, iPSC models of *MAPT* coding mutations present in ubiquitously expressed exons have given mechanistic insights into disease. R406W mutation neurons (together with IVS 10 + 14 neurons) exhibit increased aggregation of tau when analysed by dot-blot and conformation-specific antibodies to misfolded tau [113]. R406W and IVS 10 + 14 mutation neurons were also more susceptible to calcium-induced cell death, a feature reversed via inhibition of calcium influx [113].

In spite of a predominance of 3R tau, successful modelling of the exon 10-located mutation P301L has also been demonstrated. An early electrical maturation was observed in neurons bearing this mutation, similar to that seen in N279K neurons. This is particularly interesting given that the relative expression of mutant tau would be quite low and suggests that just a small amount of mutant tau protein may be able to induce FTD-associated cellular phenotypes [112].

Modelling tau aggregation in vitro has been a longstanding challenge, in part due to its high solubility under physiological conditions. A notable absence from iPSC-neurons is the presence of bona fide tau tangles. This is perhaps unsurprising given the solubility of tau protein and the relatively short time periods iPSC-neurons are cultured for. Verheyen and colleagues were able to induce tau aggregation in iPSC-neurons using transfection of pre-assembled fibrils [117]. Further, silver-positive, insoluble tau aggregates were generated in a 3D model of AD, although it should be noted this was in the context of APP and PSEN1 overexpression [79].

The tau transmission hypothesis was also studied using control iPSC-derived neurons and exogenous tau species [118]. Oligomeric tau species were able to template endogenous tau to form insoluble aggregates, leading to neurite retraction, synaptic loss and calcium signalling imbalance. Importantly, these features were particular to oligomeric tau and not monomeric tau, hinting at the toxic species in vivo. Reinforcing this, iPSC-neuronal conditioned media was shown to contain tau in an activity-dependent manner [119] and extracellular tau was shown to be taken up by recipient neurons, demonstrating an ability of tau to be transmitted between neurons.

#### Modelling tau risk variants in iPSC-neurons

Recently, the tau A152T mutation was identified as a rare variant that increases risk of tauopathies within the FTD spectrum [120–122]. The molecular mechanisms linking this variant to neurodegeneration have been investigated using heterozygous and homozygous iPSC, both patient-derived and genome-engineered. This work has revealed a variety of cellular phenotypes, including an upregulation in tau protein associated with A152T, demonstrated by western blotting and mass spectrometry [123]. This is accompanied by an increase in tau phosphorylation at multiple disease-associated epitopes and a decrease in tau solubility. Importantly, in spite of these changes, neurons were negative for Thioflavin S, supporting the idea that iPSC-neurons model pre-tangle changes to tau. A separate study showed that A152T neurons, together with 10 + 16 FTD neurons, display increased levels of the matrix metallo-proteases MMP2 and MMP9 as a result of activation of the ERK pathway [124]. Further, inhibition of MEK, the upstream activator of ERK, reduced MMP9 expression and alleviated neuronal death [124].

### Understanding tau–Aβ interactions

It has proven difficult to generate in vivo models that recapitulate the formation of plaques, tangles and neuronal death, with rodent models typically relying on the overexpression of multiple mutant genes to generate both pathologies [125]. iPSC-neurons therefore provide an attractive tool to dissect tau–Aβ interactions in a human neuronal system. As discussed earlier, iPSC-neurons from fAD patients show increased levels of phosphorylated tau [123], and two groups have shown a link between APP mutations and higher tau protein levels, suggesting a previously unidentified link between APP metabolism and tau proteostasis [71, 75]. Although tau hyperphosphorylation, increased insolubility and mislocalisation have all been described in iPSC-neurons, the development of tau silver-positive filaments has only been achieved in the context of overexpressed mutant APP and PSEN1 [79].

Intriguingly, a recent report generated chimeric models of human neurons transplanted into an AD mouse model to investigate the susceptibility of human cells to Aβ. The human neurons showed significant neurodegeneration, in contrast to mouse neurons, which were unaltered in number. Interestingly, the authors detected early markers of tau pathology such as MC1 immunoreactivity, but no overt tangle formation, suggesting a dissociation between tangles and neuronal death as suggested previously [99, 100, 116].

It remains be determined whether the crucial role of tau in neurodevelopment will protect immature neurons from the development of all features of tau pathology. The use of gene editing technologies such as CRISPR will permit the development of tau knockout iPSCs to test whether tau is essential to amyloid toxicity in human neurons, as has been shown previously in animal models [126, 127].

### Non-neuronal cell types

This review has focussed predominantly on neuron and astrocyte models of AD as these are the cell types that have been most extensively studied to date. However, it is clear that other cell types are implicated in pathogenesis, most notably microglia for their the role together with neuroinflammation in AD risk and disease progression. The identification of genetic polymorphisms in genes enriched in microglia (brain resident macrophages) provides compelling evidence for the involvement of this cell type in AD pathogenesis.

Rare mutations in the TREM2 gene lead to the bone condition Nasu Hakola disease. Heterozygous mutations, however, have been shown to increase AD risk by one-third [89]. It is believed that the cell surface TREM2 protein functions in phagocytosis and cell signalling. Work on the molecular basis of disease is on-going.

Genome-wide association studies have also identified polymorphisms in CR1, CD33 and MS4A, genes enriched in microglia, that increase risk of AD [128]. In addition to genetic evidence, an increase in activated microglia is observed in AD pathological tissue surrounding amyloid plaques, and experimental evidence showing microglial ablation halts tau propagation [129]. Thus, the development of human iPSC-derived microglial models is warranted to further understand the contribution of this cell type to disease [130].

Initial studies are now being reported demonstrating iPSC-derived microglial-like cells [131, 132]. Due to the fact that microglia develop through the haematopoietic lineage and migrate into the neural tube before the blood–brain barrier forms, specific differentiation strategies are required. After the specification of haematopoietic progenitors expressing CD34 and CD43, granulocyte macrophage colony stimulating factor (GM-CSF) triggers microglial-like specification and expression of markers such as IBA1 and CD11b. iPSC-derived microglia respond to Aβ and oligomeric tau by upregulating AD-associated genes, such as TREM2, CD33, MS4A and APOE [133].

Impairment of the blood–brain barrier has also been implicated in AD progression [134]. The generation of vascular endothelial cells and pericytes from iPSCs would aid our understanding of the blood–brain barrier in AD and protocols to develop these cell types have also progressed in recent years [135, 136]. Together with astrocytes and neurons, pericytes and endothelial cells have been shown to generate a blood–brain barrier model amenable to drug permeability screening [137]. The development of complex models, incorporating multiple cell types in isolation and combination, will be important for our understanding of AD mechanisms.

Due to the diverse developmental origins of microglia, pericytes and endothelial cells, these lineages are not present in 3D cortical organoid cultures. Of these cell types, iPSC-derived microglia have been added to 3D cultures, showing integration, migration and maturation [131, 133].

### Challenges

#### Addressing foetal characteristics in iPSC-derived neurons

A major drawback of using iPSC-derived neurons for AD research (in both 2D and 3D paradigms) is represented by the relative developmental age of the neurons. As differentiation pathways mirror developmental timing, 100-day protocols generating cortical neurons in vitro represent a foetal developmental stage [26], as exemplified by tau splicing mentioned above [110–113]. This represents a significant hurdle when investigating diseases where the single most significant risk is ageing and whereby disease onset typically initiates after the fifth decade of life.

One challenge is the neuronal versus glia bias due to the later stage gliogenic switch [24]. As a consequence, in vitro cultures are not representative of brain due to a reduced proportion of astrocytes [138]. This will have implications on neuronal maturity [139] as well as non-cell autonomous disease mechanisms (for a review of astrocytes’ role in AD see [140]).

A handful of studies have now attempted to address this. The first used overexpression of the mutant Lamin A gene, known to lead to the premature ageing disease progeria [114]. Progerin overexpressing iPSC-derived dopaminergic cells expressed epigenetic marks of ageing and displayed hallmarks of Parkinson’s disease that were absent in control cells. The second study made use of direct reprogramming techniques, whereby fibroblasts are transdifferentiated into neurons without passing through a proliferative stage [141]. This process lacks the pluripotent stage and so erasure of epigenetic marks does not occur. For this reason, when iNs (induced neuron) were compared to iPSC-derived neurons, genetic signatures of ageing were maintained and ageing-associated reductions in nuclear–cytoplasmic transport were evident. Pharmacological inhibition of telomerase during stem cell maintenance and early differentiation time points was also shown to increase aging features and disease-associated marks in iPSC-derived neurons [115], including reduced dendrite number, increased DNA damage and increased ROS.

Concurrent with inducing ageing in cells in culture, cell proliferation can be manipulated and inhibited. Strategies such as inhibition of Notch signalling can prevent cell division and initiate terminal differentiation [44]. These strategies shorten differentiation protocols, however, as they rely on inhibition of γ-secretase, and careful consideration is required in an AD research setting. In 3D, these strategies are yet to be fully investigated, although initial reports suggest Notch inhibitors can enhance neuronal maturity [45].

These studies have been performed on 2D cultures but could also be applied to 3D cultures. However, this will be more technically challenging due to the inability to treat all cells within the 3D structure. Studies such as these will help to tease apart the deficiencies that underlie disease mechanisms and the ageing process that leads to a breakdown of cell coping pathways, two processes which when combined lead to neurodegeneration.

#### Variability—gene editing

Variability between iPSC clones from the same individual, and iPSCs from different individuals, remains the major hindrance of iPSC technology (reviewed in [142]). Generating enough statistical power through numbers of cell lines is problematic. Therefore, careful selection of control lines to which patient-derived cultures can be compared is crucial. The use of unaffected siblings or family members as control lines goes some way to reduce the variable genetic landscape between cell lines in a given study via reducing allelic discrepancies. Indeed, several studies have been able to employ cell lines from discordant (affected versus unaffected) monozygotic twins in iPSC-neurodegenerative research [143–145]. The benefit of such studies is that the genome is identical except for the changes in disease contraction.

Making use of the two cell endogenous repair pathways for DNA lesions, namely non-homologous end joining (NHEJ) and homology directed repair (HDR), gene editing is now possible in iPSCs (reviewed [146]). Endonucleases are targeted to specific sites in the genome via protein DNA-binding domains or guide RNAs, examples of which include zinc finger nucleases [147, 148], TALENs [149] and CRISPR/Cas9 [150, 151]. The CRISPR/Cas9 system benefits from being encoded by short guide RNAs rather than engineered proteins, simplifying experimental design considerably.

Subsequent cleavage events via the fused endonuclease can then lead to either incomplete repair by NHEJ, leading to gene knockout via frameshift mutations, or repair by HDR via template DNA supplied to the reaction to incorporate the intended genome changes. The first strategy leads to knockouts with relative ease, whereas the second can repair mutations or incorporate new tags, mutations or longer coding cassettes with lower efficiency. Several groups have already harnessed this approach to generate isogenic dementia mutation lines [67, 76, 79, 152], and genome engineering will be particularly powerful for the assessment of genetic risk variants, where multiple genes of low effect can be engineered in a controlled way on an isogenic background.

These techniques enable the generation of isogenic controls, whereby the genomic landscape of control and test iPSC lines are identical except for the mutation or knockout of interest. These technologies will go a long way to reduce variability and will become more and more commonplace in iPSC-research into neurodegeneration as the technology becomes more accessible.

### Future perspectives and challenges

iPSC-derived neurons offer a revolutionary new angle on research into dementia. iPSCs offer a limitless supply of human neurons that are electrically active and represent the cell type that degenerates early in AD. The fact that these cells are morphologically, electrically and transcriptionally similar to human cortical neurons in the brain offers huge benefits to AD research. Additionally, as the iPSCs are patient-derived, mutations, SNPs and CNVs can be studied in the neurons in vitro. It remains difficult to model sAD via this approach; however, similar pathways are being implicated in multiple studies, including enlarged endosomes, ROS damage and synaptic toxicity.

A number of drawbacks are present to the field. Variability between cell lines necessitates large numbers of independent replicates, both biological and experimental. The gene-editing technologies now available to researchers offer a solution to this, although this is still a non-trivial first technical step to a project. The other major drawback is biological ageing, whereby an ageing setting is hard to recapitulate in vitro. This remains a major hurdle to the field; however, it can be turned into a strength as the underlying cellular deficiencies can be studied free from widespread changes secondary to the disease-initiating factors. Specifically, the direct effects of mutations and risk variants can be investigated free from late-stage, complex changes that result from a devastating cellular environment of neurodegeneration. These underlying pathways—which only manifest in an aging setting due to reduced cellular coping mechanisms or an accumulation of damage—can therefore be investigated directly and may become the basis of strategies to reverse degeneration early in disease progression.

The use of 3D iPSC-derived neuronal cultures will certainly complement the field. Some pathological changes are now shown to be higher in 3D cultures compared to 2D cultures, and the complexity of 3D organoids (both in tissue architecture and cell types) will add insights into complex events such as spread of pathology and cell behaviour.

The benefits of both 2D and 3D platforms have been shown in the field of drug discovery, whereby responses to β- and γ-secretase inhibitors, as well as other drugs, have been shown to impact APP processing and downstream effects on tau. These platforms may prove invaluable in future in vitro screens prior to human trials, especially for novel pathway hits, and may even be used to stratify patients’ personalised medicine.

## Conclusions

The AD field is set for an explosion of new information gleaned from stem cell biology. Understanding the earliest events in dementia and the direct molecular effects of fAD mutations and genetic risk variants and providing a human neuronal platform for drug screening are strengths that iPSC technology can add to the field. Although several hurdles remain, in concert with other models of AD, iPSC models look set to make a valuable contribution to efforts to develop novel treatments for AD.

## Abbreviations

2D: Two-dimensional
3D: Three-dimensional
AD: Alzheimer’s disease
APP: Amyloid precursor protein
Aβ: Amyloid beta
BDNF: Brain-derived neurotrophic factor
BFCN: Basal forebrain cholinergic neuron
CNV: Copy number variation
EB: Embryoid body
ER: Endoplasmic reticulum
fAD: Familial Alzheimer’s disease
FTD: Frontotemporal dementia
HDR: Homology directed repair
hESC: Human embryonic stem cell
iPSC: Induced pluripotent stem cell
NHEJ: Non-homologous end joining
NSAID: Non-steroidal anti-inflammatory drug
PSEN: Presenilin
pTau: Phosphorylated tau
ROS: Reactive oxygen species
sAD: Sporadic Alzheimer’s disease
sAPP: Soluble APP
SNP: Single nucleotide polymorphism
tTau: Total tau.
